# The vibration response mechanism of a blade disk rotor system under the coupling effects of cracks and aerodynamic forces

**DOI:** 10.1038/s41598-022-05543-x

**Published:** 2022-01-27

**Authors:** J. Yang, J. Xie, T. Wang, F. Yang, J. Chen

**Affiliations:** 1 School of Traffic and Transportation Engineering, Central South University, Changsha, 410075 People’s Republic of China; 2 Kunming Institute of Physics, Kunming, 650000 People’s Republic of China; 3 State Key Laboratory for Manufacturing Systems Engineering, Xi’an Jiaotong University, Xi’an, 710049 People’s Republic of China

**Keywords:** Aerospace engineering, Mechanical engineering

## Abstract

The important role of a dynamic model is to study the response characteristics of a system under different parameters or fault states. These response characteristics can be used in many aspects, such as condition monitoring and fault diagnosis. Usually, the response characteristics can be obtained through numerical analysis, but we do not know why such characteristics appear, which hinders our understanding and utilization of vibration. The innovation of this paper is to reasonably explain why such response characteristics appear**.** First, a simplified dynamic model of a typical blade disk rotor system is constructed by using the classical continuous parameter modeling method. Based on the dynamic model, for two structural forms of moving and stationary blades, the typical characteristics of the vibration response under the actions of aerodynamic force and blade cracks are analyzed by means of numerical solution. Then, from the perspective of kinematics and dynamics, the internal mechanism between the vibration responses and the excitations is revealed. Finally, based on Number Theory, the response characteristics and mechanisms of typical structures are summarized, and the general laws of responses with general structural forms are established.

## Introduction

The mechanism of vibration response characteristics and fault characteristics is the key priori information for equipment vibration phenomenon analysis, health assessment and fault diagnosis. Therefore, the revelation of the vibration response mechanism has been a basic scientific problem that experts are committed to solving for a long time. After continuous research, scholars have comprehensively and accurately revealed the vibration characteristics and response mechanisms of key parts such as shafts, bearings and gears.

The vibration characteristics of amplitude and phase are used to detect the imbalance of a rotor system^1^. According to the characteristics and mechanism of rotor imbalance, to suppress rotor vibration caused by imbalance, an active control method using a magnetic actuator has been proposed^2^. Xie^3,4^ discovered a new modulation frequency characteristic under the disturbance state of a cracked rotor system and explained the modulation mechanism. The instantaneous whirling speed was defined, and its response mechanism was revealed. Through dynamic analysis and experimental research, the mechanism of torsional vibration characteristics of cracked and noncracked rotor systems was discussed^5^. Li^6^ used the zero-stress intensity factor method to solve the stiffness of a rotor system with slant cracks and studied the effects of fractional order, speed and crack depth on the dynamic characteristics of a rotor system. The rotating orbit, time domain response and spectrum were obtained to show the phenomenon of superharmonic resonance in a hollow shaft cracked rotor system^7^. Cao^8^ discussed the contact characteristics between a roller and race ways and the changes in roller rotation angular speed. Xiang^9^ proposed a nonlinear dynamic model of bearings based on a collision system to simulate the vibration characteristics of different fault types. Guo^10^ proposed a new dynamic model and studied the double pulse behavior and mechanism of bearing raceway surface spallation. Bachar^11^ studied the influence of working conditions and surface roughness on the vibration characteristics of a spur gear transmission and studied the detection ability of a single tooth surface fault. Chen^12^ analyzed the changes in dynamic responses in the time and frequency domains for different wear degrees at the tooth surface. Yang^13^ revealed the variation law of the time-varying meshing stiffness, the time history and the frequency spectrum of vibration signals under chipping damage. Cao^14^ discussed the effects of external load and damping parameters on the frequency response and force response curve by using an equivalent nonlinear model of a nonlinear beam truss.

The above mechanism-revealing research has significantly promoted the mastery of the vibration law of key parts. Based on these efforts, many reliable and accurate fault diagnosis methods have been established, which has important theoretical significance and economic value.

A blade disk rotor system is different from the above typical parts and is composed of shafts, disks and multiple blades. Scholars have studied the vibration characteristics and mechanisms from the aspects of single-blade, blade-disk, and blade-disk-rotor systems.

Zhao^15,16^ investigated a coupled modeling method and free vibration characteristics of a graphene nanoplatelet (GPL) reinforced blade-disk rotor system in which the blade has a pre-twist angle and setting angle and presented an investigation on nonlinear forced vibration characteristics of a spinning shaft-disk assembly resting on sliding bearing supports. Yang^17,18^ investigated the free vibration of functionally graded (FG) pre‐twisted blade‐shaft/disk-shaft rotor system reinforced with GPLs. Wang^19,20^ investigation on vibration characteristics of GPL reinforced disk-shaft rotor with eccentric mass resting on elastic supports and presented the theoretical modeling of a functionally graded GPL-reinforced assembled beam–plate structure resting on elastic supports. Wu^21^ indicated that when the excitation frequency changes from 0 to the first resonance frequency of a cracked beam, the crack breathing frequency increases linearly, and the crack breathing frequency changes nonlinearly with a further increase in the excitation frequency. Yang^22,23^ found that severe cracks are expected to seriously reduce the stiffness of rotating blades and significantly reduce the resonance frequency. Li^24^ investigated the effects of the thickness-taper ratio, pre-twist angle, rotational speed, and connection stiffness on blade modal characteristics. Zi^25^ indicated that the complexity of the natural frequency and forced response depends on the length and relative position of cracks. When cracks or detuning occur in an impeller, the response amplitude of the blade fluctuates periodically with the number of blades. Joachim^26^ evaluated the effect of small detuning on the vibration amplitude of a 2D blade disk. Heydari^27^ studied the effects of the blade stagger angle and pre-twist angle on shaft-bending and blade-bending coupling vibration. Jin^28^ revealed the nonlinear vibration characteristics caused by blade-casing rubbing of a real dual rotor aeroengine. Ma^29,30^ showed that an original hardening type nonlinearity may be enhanced or transformed into a softening type due to the nonlinear stiffness of a bearing, and the rubbing dynamic responses of the shaft-blisk-casing system at different speeds, disc imbalance, disc position and speed are solved. Liu^31^ studied the dynamic behavior of casing acceleration of the whole aeroengine from the aspect of blade-casing rubbing fault diagnosis. Wei^32^ showed that under the excitation of a blade loss load, the transient response of the system has obvious impact characteristics, and the stiffness and damping of the rear bearing of the fan have a significant impact on the transient response. Zhao^33,34^ used the elastic supported coupling finite element model to study the rubbing of a mistuned blade disk system with variable thickness blades and showed that the cracks significantly exacerbated the vibration of the blades. A large number of research results have been obtained in the characteristic analysis of blade disk rotor systems. However, the current research shows that different structures have different characteristics, the universality law has not been established, and the deeper mechanical essence mechanism between characteristics and excitation is not clear.

It is of great significance and a great challenge to analyze the essential mechanism and establish the general law of response characteristics of the bladed disk rotor system under typical excitation. In this paper, some of the work will be carried out tentatively, and only the response mechanism and law of a circularly symmetric bladed disk rotor system (structural detuning is not considered) under the coupling effects of aerodynamic forces and cracks will be studied.

This paper is arranged as follows. A simplified dynamic model of a typical blade disk rotor system is constructed in “Vibration model of blade disk rotor system” to provide the basis for response characteristic analysis. The typical characteristics of the vibration response under aerodynamic loading and blade cracks for two structural forms are analyzed in “Numerical analysis of response characteristics”. Finally, in “Mechanism revealing and law explorating of vibration response”, the response characteristics and mechanism of typical structures are summarized, and general laws with general structural forms are obtained. The conclusions are drawn in “Conclusion”.

## Vibration model of blade disk rotor system

This paper focuses on the response characteristics and response mechanisms of a blade disk rotor system excited by the aerodynamic force between the moving and stationary blades and the stiffness parametric excitation of the blade cracks. Therefore, the model construction in this section adopts the common and classic continuous parameter modeling method to establish a simplified vibration model of a single-stage blade disk rotor system. The aerodynamic excitation between moving and stationary blades is simulated by typical traveling waves, and the stiffness parameter excitation of cracks is simulated by the stiffness breathing function published in our last paper.

### Modeling of normal system

The continuous parameter modeling method is a common and mature modeling method that has been widely used by scholars^31,35,36^. Based on this method, we have established the vibration model of a single-stage and a three-stage blade disk rotor system in our recent publications^37,38^. Therefore, for the simplified model of the single-stage blade disk rotor system required in this paper, we will not give the proof of the formula in detail but only introduce the basic modeling process.

#### Geometric model

**Figure 1:**
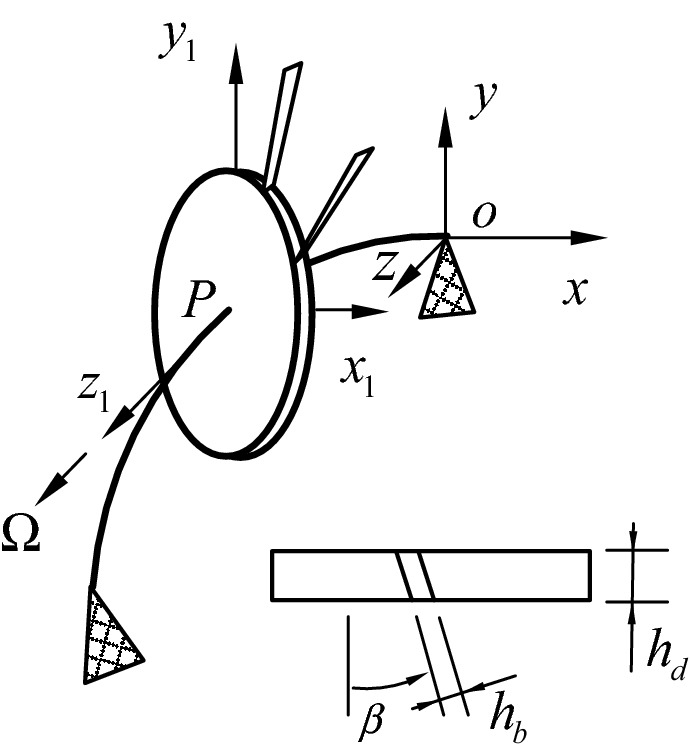
Schematic diagram of multistage bladed disk rotor system.

Figure 1 is the sketch map of the blade disk shaft system, and contains a shaft with bending and torsional degree of freedoms, a disk which can be treated as a rigid body, and some flexible blades fixed onto the outer edge of the disk with a setting angle *β*. *0xyz, Px*_1_*y*_1_*z*_1_, *Ω, h*_*d*_ and *h*_*b*_ are the global coordinate, the rotating coordinate, the rotational speed, the thicknesses of the disk and the thicknesses of blade, respectively.

**Figure 2:**
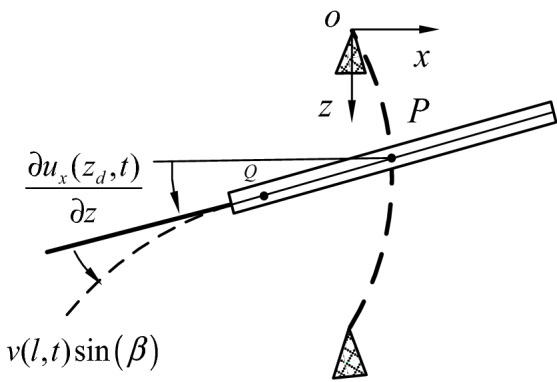
Motion decompositions in the *ozx* plane.

**Figure 3:**
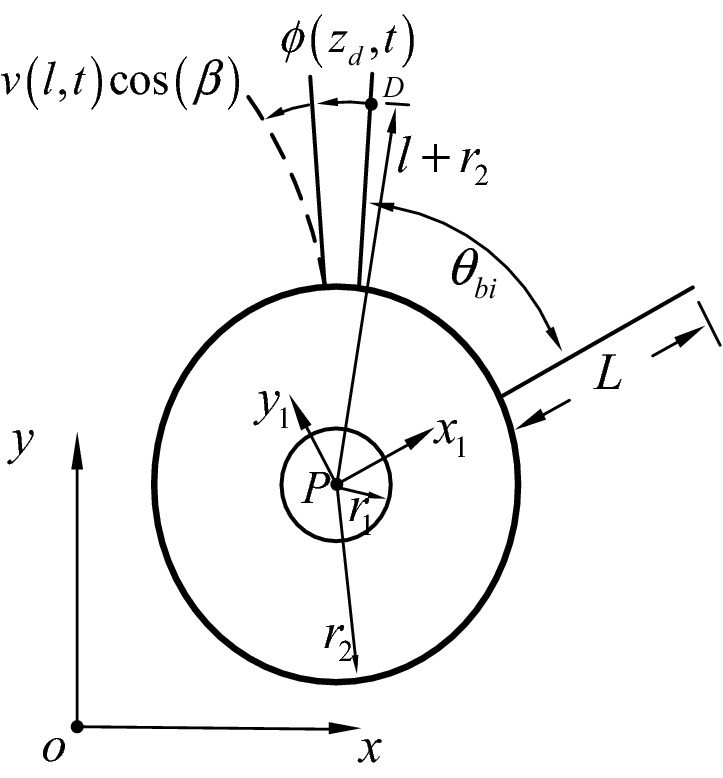
Motion decompositions in the *oxy* plane.

Figures 2 and 3 show the motion decompositions of the blade-disk-shaft system in the *oxy* and *ozx* planes. In this paper, the disk is assumed to be a rigid body without considering its elastic deformation. The thick line and dashed line represent the rigid body displacements and the elastic deformations, respectively.

Figure 2 shows a schematic diagram of the motion decomposition in the *ozx* plane. Q, *u*_*x*_(*z*, *t*), $\frac{{\partial u_{x} (z_{d} ,t)}}{\partial z}$ and *v*(*l*, *t*) sin (*β*) denote the arbitrary micro-unit in the disk, the deflection of the shaft in x-direction, the swing angle at *z*_*d*_, the out-plane deflection of blades, respectively.

Figure 3 shows a schematic diagram of the motion decomposition in the *oxy* plane. *D, L, r*_1_, *r*_2_, $\phi (z_{d} ,t)$, $v(l,t)\cos \;(\beta )$ denote the arbitrary microunit in the blade, the length of the blade, the inner diameter of the disk, the outer diameter of the disk, the torsional displacement, the in-plane deflection of blade, respectively. *l* represent the distance of *D* from the root of the blade*.*

The coordinates of the arbitrary microunit in the shaft with, the arbitrary microunit Q in the disk and the arbitrary microunit D in the blade are represented as $u = \left( {u_{x} (z),u_{y} (z),z} \right)$, (*x*_*Q*_, *y*_*Q*_, *z*_*Q*_), (*x*_*D*_, *y*_*D*_, *z*_*D*_), respectively. The detailed expressions of the absolute coordinates for Q and D are shown in ESM Appendix A.

#### Energy equations

The velocity is the derivation of the displacement and the kinetic energy is the integration about the velocity. Therefore, the total kinetic energy of the shaft is

1 $$ T_{s} = \frac{1}{2}\rho_{s} A_{s} \int_{0}^{s} {(\dot{u}_{x}^{2} + \dot{u}_{y}^{2} )dz + } \frac{1}{2}\rho_{s} I_{sp} \int_{0}^{s} {(\Omega + \dot{\phi })^{2} {\text{d}} z} $$

where *S, ρ*_s_, *A*_s_, *I*_*sp*_ denote the length of the shaft, the density, the area of the shaft cross section, the polar moment of inertia, respectively, and $I_{sp} = \int_{{A_{s} }} {r^{2} dA} = \frac{\pi }{2}r_{s}^{4}$.

According to the references^39,40^, the potential energy of the shaft can be expressed as follows:

2 $$ U_{s} = \frac{1}{2}EI_{sx} \int_{0}^{s} {\left( {\left( {\frac{{\partial^{2} u_{x} }}{{\partial z^{2} }}} \right)^{2} + \left( {\frac{{\partial^{2} u_{y} }}{{\partial z^{2} }}} \right)^{2} } \right){\text{d}} z + } \frac{1}{2}G_{s} I_{sp} \int_{0}^{s} {(\frac{\partial \phi }{{\partial z}})^{2} {\text{d}} z} $$

where *E*_s_, *G*_s_ and *I*_*sx*_ are the Young’s modulus, the shear modulus, the area moment, respectively, and $I_{sx} = \int_{{A_{s} }} {y^{2} dA} = \frac{\pi }{4}r_{s}^{4}$.

Similar to the shaft, the kinetic energy of the disk can be expressed as follows:

3 $$ T_{d} = \frac{1}{2}\rho_{d} h_{d} \int\limits_{{r_{1} }}^{{r_{2} }} {\int\limits_{o}^{2\pi } {\left( {\dot{x}^{2}_{Q} + \dot{y}^{2}_{Q} + \dot{z}_{Q}^{2} } \right)r{\text{d}} r{\text{d}} \theta } } $$

and the kinetic energy of the *i*th blade can be expressed as follows:

4 $$ T_{bi} = \frac{1}{2}\rho_{b} A_{b} \int\limits_{0}^{L} {\left( {\dot{x}^{2}_{D} + \dot{y}^{2}_{D} + \dot{z}^{2}_{D} } \right)} {\text{d}} l $$

where *A*_b_ is the area of the blade cross section.

According to the reference^33^, the bending and centrifugal potential energy of the *i*th blade can be given as follows:

5 $$ \begin{aligned} U_{bi} & = \frac{1}{2}EI\int\limits_{0}^{L} {\left( {\frac{{\partial^{2} v_{i} (l,t)}}{{\partial r^{2} }}} \right)^{2} } {\text{d}} l \\ & \quad { + }\frac{1}{4}\rho A_{b} \Omega^{2} \int\limits_{0}^{L} {\left[ {(r_{2} + L)^{2} - \left( {r_{2} + l} \right)^{2} } \right]\left( {\frac{{\partial v_{i} (l,t)}}{\partial l}} \right)^{2} {\text{d}} l} \\ \end{aligned} $$

#### Assumed modes

The continuous system is discretized by the assumed mode method, in which the displacements of the shaft is discretized as follows:

6 $$ u_{x} (z,t) = {\mathbf{U}}^{T} {\mathbf{q}}_{x} = {\mathbf{q}}_{x}^{T} {\mathbf{U}} $$

7 $$ u_{y} (z,t) = {\mathbf{U}}^{T} {\mathbf{q}}_{y} = {\mathbf{q}}_{y}^{T} {\mathbf{U}} $$

8 $$ \phi (z,t) = {{\varvec{\Phi}}}^{T} {\mathbf{q}}_{\phi } = {\mathbf{q}}_{\phi }^{T} {{\varvec{\Phi}}} $$

and, the displacement of blades is discretized as follows:

9 $$ v(l,t) = {\mathbf{V}}^{T} {\mathbf{q}}_{v} = {\mathbf{q}}_{v}^{T} {\mathbf{V}} $$

where **U**, **Φ**, **V** denote the assumed modal matrices, **q**_*x*_, **q**_*y*_, **q**_*ϕ*_ and **q**_*v*_ denotes the generalized coordinates.

#### Discrete vibration equation

Bringing these displacements which discretized by assumed modes into the energy expressions, then employing the Lagrange equations (10, 11), the discretized equations of motion in matrix notation can be obtained in Eq. (12):

10 $$ \frac{d}{dt}\left( {\frac{\partial L}{{\partial {{\varvec{\upeta}}}}}} \right) - \frac{\partial L}{{\partial {{\varvec{\upeta}}}}} = {\mathbf{Q}} $$

11 $$ L = T - U = T_{s} - U_{s} + \sum\limits_{j = 1}^{n} {T_{dj} + T_{Bj} - U_{Bj} } $$

12 $$  {\mathbf{M}}  {\varvec{ \ddot{\eta } }}  +  {\mathbf{C}} {\varvec{ \dot{\eta } }} +  {\mathbf{K}}  {\varvec{ \eta  }}  = Q   $$

where **η** denotes the generalized coordinate matrix, and **Q** is the generalized force matrix corresponding to the generalized coordinates, and the detail formula is shown in ESM Appendix B.

### Modeling of aerodynamic force

**Figure 4:**
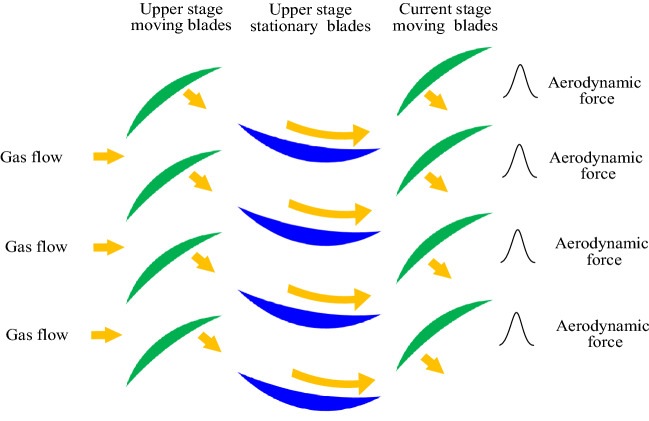
Schematic diagram of aerodynamic force between moving blade and stationary blade.

The working mode of the blade disk rotor system is compressed gas or driven by gas. As shown in Fig. 4, according to the working principle of the blade disk rotor system, each time the moving blade passes through a stationary blade, it will be coupled with the gas flow through the upper stationary blade, to form a pulse aerodynamic force on the moving blade.

Based on the principle of Fourier decomposition, the pulse signal can be decomposed into the superposition of sinusoidal signals with the pulse frequency as the fundamental frequency^41,42^. Therefore, the aerodynamic force on the moving blades *v*_i_ caused by the gas of the upper stationary blade can be qualitatively modeled as:

13 $$ f_{{v_{i} }} (l,t) = p_{0} + \sum\limits_{{K_{j} = 1}}^{{K_{j} }} {p_{{K_{j} }} \sin \left( {K_{j} N_{s} \Omega t + \varphi_{i} } \right)} $$

where, *p*_0_ is the constant component; $p_{{K_{j} }}$ is the components of the *K*_*j*_-th frequency; *K*_*j*_ is the order of frequency and *K*_*j*_ is the 1,2,3,4; *φ*_*i*_ is the initial phase of the *i*-th blade; $N_{s} \Omega$ is the fundamental frequency of pulse; *N*_*s*_ is the number of stationary blades.

### Modeling of blade crack stiffness parameter excitation

**Figure 5:**
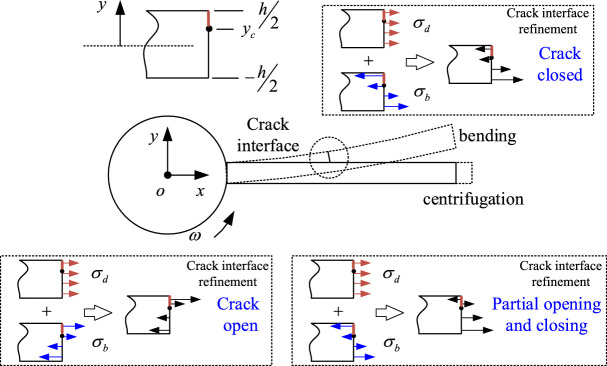
Schematic diagram of crack breathing in a rotating blade.

Under the action of centrifugal stress caused by rotation and bending vibration caused by aerodynamic force, breathing effects appear on the contact surface of crack as shown in Fig. 5, so as to transiently change the stiffness of the cracked blade. Therefore, stiffness parameter excitation arises in the system.

The method adopted to model the cracked blade is from our recent publication^43^. In the modeling of the cracked blade, the released energy associated with the crack is considered as follows:

14 $$ U_{c} = \frac{1}{2}\frac{{(EI)^{2} }}{{K_{{{\text{crack}}}} }}{{\varvec{\upeta}}}^{T} {\varvec{V}}(l_{c} )^{\prime\prime}{\varvec{V}}(l_{c} )^{\prime \prime T} {{\varvec{\upeta}}} $$

where *K*_*crack*_ is the breathing stiffness of the crack, depending on the vibration response (bending stress *σ*_*b*_) and the centrifugal effect (centrifugal stress *σ*_*d*_). The breathing function is:

15 $$K_{{{\text{crack}}}}  = k_{c}  \times \left\{ {\begin{array}{*{20}l}    \infty  \hfill & {\sigma _{b}  \ge {\raise0.7ex\hbox{${\sigma _{d} }$} \!\mathord{\left/ {\vphantom {{\sigma _{d} } {y_{c} }}}\right.\kern-\nulldelimiterspace} \!\lower0.7ex\hbox{${y_{c} }$}}} \hfill  \\    {\left( {\frac{{h/2 - y_{c} }}{{y_{0}  - y_{c} }}} \right)^{3} } \hfill & {{{\sigma _{d} } \mathord{\left/ {\vphantom {{\sigma _{d} } {\left( {{\raise0.7ex\hbox{$h$} \!\mathord{\left/ {\vphantom {h 4}}\right.\kern-\nulldelimiterspace} \!\lower0.7ex\hbox{$4$}} + {\raise0.7ex\hbox{${y_{c} }$} \!\mathord{\left/ {\vphantom {{y_{c} } 2}}\right.\kern-\nulldelimiterspace} \!\lower0.7ex\hbox{$2$}}} \right)}}} \right. \kern-\nulldelimiterspace} {\left( {{\raise0.7ex\hbox{$h$} \!\mathord{\left/ {\vphantom {h 4}}\right.\kern-\nulldelimiterspace} \!\lower0.7ex\hbox{$4$}} + {\raise0.7ex\hbox{${y_{c} }$} \!\mathord{\left/ {\vphantom {{y_{c} } 2}}\right.\kern-\nulldelimiterspace} \!\lower0.7ex\hbox{$2$}}} \right)}} < \sigma _{b}  < {\raise0.7ex\hbox{${\sigma _{d} }$} \!\mathord{\left/ {\vphantom {{\sigma _{d} } {y_{c} }}}\right.\kern-\nulldelimiterspace} \!\lower0.7ex\hbox{${y_{c} }$}}} \hfill  \\    1 \hfill & {\sigma _{b}  \le {{\sigma _{d} } \mathord{\left/ {\vphantom {{\sigma _{d} } {\left( {{\raise0.7ex\hbox{$h$} \!\mathord{\left/ {\vphantom {h 4}}\right.\kern-\nulldelimiterspace} \!\lower0.7ex\hbox{$4$}} + {\raise0.7ex\hbox{${y_{c} }$} \!\mathord{\left/ {\vphantom {{y_{c} } 2}}\right.\kern-\nulldelimiterspace} \!\lower0.7ex\hbox{$2$}}} \right)}}} \right. \kern-\nulldelimiterspace} {\left( {{\raise0.7ex\hbox{$h$} \!\mathord{\left/ {\vphantom {h 4}}\right.\kern-\nulldelimiterspace} \!\lower0.7ex\hbox{$4$}} + {\raise0.7ex\hbox{${y_{c} }$} \!\mathord{\left/ {\vphantom {{y_{c} } 2}}\right.\kern-\nulldelimiterspace} \!\lower0.7ex\hbox{$2$}}} \right)}}} \hfill  \\   \end{array} } \right.$$

where *k*_*c*_ is the stiffness of the open crack, and $k_{c} = \frac{EI}{{6\left( {1 - \mu^{2} } \right)hQ(\gamma )}}$, and *γ* denote the relative crack depth^44^.

## Numerical analysis of response characteristics

Based on the dynamic model, special case studies for two structural forms of moving and stationary blades are explored by numerical solutions, to intuitively present the expression of typical response characteristics under aerodynamic force and crack stiffness parameter excitations.

### Response characteristics of synchronous excitation

In a typical simplified system with five stationary blades (*N*_s_ = 5) and five moving blades (*N*_d_ = 5), the five moving blades are synchronously excited by the aerodynamic force (synchronous excitation).

This paper focuses on the qualitative study of the characteristics and mechanism under aerodynamic loading and crack excitation. Therefore, only the frequency components of transverse vibration and torsional vibration are compared and analyzed.

Considering the inevitable eccentricity of the system, the load forms studied in this section include: eccentricity (Ecce), aerodynamic force (AeroF), crack and aerodynamic force (Crack + AeroF), and crack, eccentricity and aerodynamic force (Crack + Ecce + AeroF).

#### Transverse vibration response

**Figure 6:**
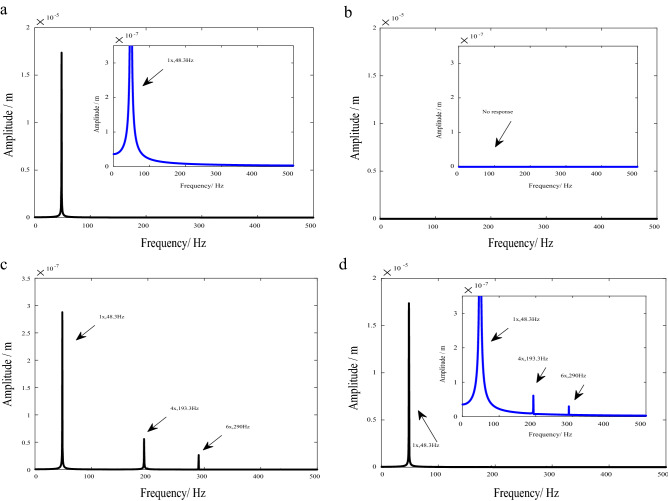
Spectrums of transverse vibration response (*N*_s_ = *N*_d_ = 5). (**a**) Ecce. (**b**) AeroF. (**c**) Crack + AeroF. (**d**) Crack + Ecce + AeroF.

Figure 6 shows the frequency spectrums of the transverse vibration responses. In this case, the rotation speed is 2900 RPM, and the corresponding rotation frequency $\omega$ = 48.3 Hz (1×).

As shown in Fig. 6a, the eccentricity will lead to a rapid increase in the amplitude corresponding to 1× frequency component in the transverse vibration response.

As can be seen in Fig. 6b, when the system is only under the excitation of aerodynamic force, without eccentricity and crack excitations, the amplitude of the transverse vibration response is zero, indicating that the resultant force of each aerodynamic force on transverse vibration is zero.

Under the excitation of aerodynamic force, each blade has bending vibration. Due to the breathing effect of cracks, the bending vibration of the cracked blade is different from that of other blades, resulting in the mistuning of the vibration coupling effect of the blades on the shaft. As shown in Fig. 6c, under the coupling action of the crack and aerodynamic force, the amplitude of frequency components such as 1× = 48 Hz, 4× = 194 Hz and 6× = 291 Hz increases, where 1× is rotation frequency $\omega$, 4× is $\left( {N_{b} { + }1} \right)\omega$, 6× is $\left( {N_{b} { - }1} \right)\omega$, and *N*_b_ = 5.

In transverse vibration, the eccentricity often dominates the vibration response, the amplitude of the 1× frequency is very large, and the response characteristics under the coupling of cracks and aerodynamic forces are easily to be submerged, as shown in Fig. 6d. However, qualitatively, the characteristics caused by the coupling of cracks and aerodynamic forces are different from those of the eccentricity.

#### Torsional vibration response

**Figure 7:**
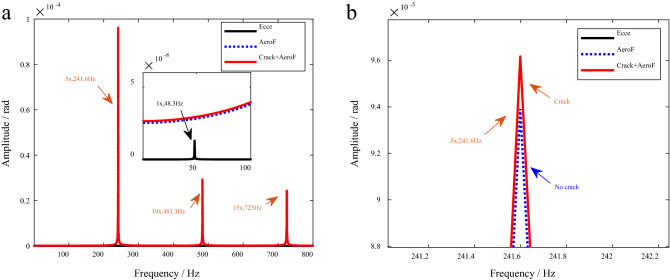
Spectrums of torsional vibration response. (*N*_s_ = *N*_d_ = 5). (**a**) AeroF. (**b**) Crack + AeroF.

Figure 7 shows the frequency spectrums of the torsional vibration responses.

As can be seen in Fig. 7, in this case, the amplitude of torsional vibration caused by bending-torsion coupling effects is very small, and it is difficult to identify the frequency components of coupled torsional vibration caused by eccentricity in the spectrum of torsional vibration response.

As shown in Fig. 7, under the excitation of aerodynamic force, the amplitude of frequency components such as 5x = 241.7 Hz, 10x = 483.3 Hz and 15x = 725 Hz increases, where 5× is *N*_b_ $\omega$, 10× is 2*N*_b_ $\omega$ and 15× is 3*N*_b_ $\omega$.

In this structural form with five stationary blades and five moving blades, the fundamental frequencies of normal and cracked blades are 5×. The crack leads to an increase in the amplitude of the 5× frequency component in the torsional vibration response, as shown in Fig. 7.

### Response characteristics of asynchronous excitation

In a typical simplified system with seven stationary blades (*N*_s_ = 7) and five moving blades (*N*_d_ = 5), the five moving blades are asynchronously excited by the aerodynamic force (asynchronous excitation), that is, each moving blade is excited by the aerodynamic force with a phase lag.

**Figure 8:**
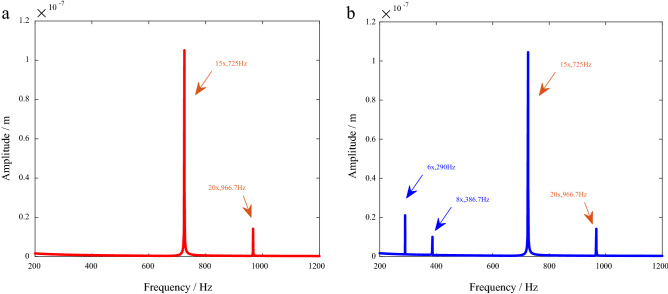
Spectrums of transverse vibration responses (*N*_s_ = 7, *N*_d_ = 5). (**a**) AeroF. (**b**) Crack + AeroF.

The transverse vibration responses under asynchronous excitation are shown in Fig. 8. There are 15× and 20× frequencies in both normal and cracked systems under aerodynamic force. When there is a crack in the blade, 6× and 8× frequency components appear in the response spectrum, but there is no such frequency component in the normal system.

**Figure 9:**
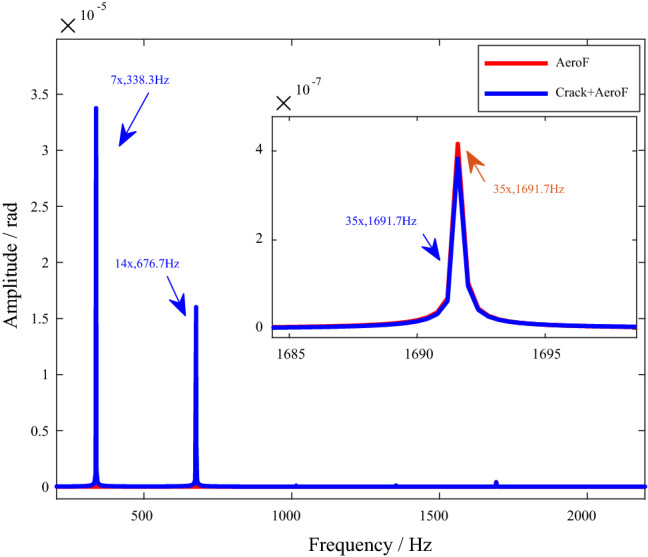
Spectrums of torsional vibration responses (*N*_s_ = 7, *N*_d_ = 5).

The torsional vibration responses are shown in Fig. 9. It can be seen in the figure that under the coupling action of aerodynamic force and cracks, there are 35× frequency components in both cracked and normal systems, indicating that the response of this frequency component is caused by aerodynamic force. However, the frequency components such as 7× and 14× appear in the cracked system, but not in the normal system. Therefore, these frequency components are caused by the stiffness parameter excitation of the crack.

In summary, special case studies based on the dynamic model show that under asynchronous excitation, the aerodynamic force leads to 15× and 20× frequency components in the transverse vibration response and a 35× ($N_{s} \times N_{d} \omega$) frequency component in the torsional vibration response. The stiffness parameter excitation of the blade crack leads to frequency components such as 6× ($\left( {N_{s} - 1} \right)\omega$) and 8× ($\left( {N_{s} + 1} \right)\omega$) in the transverse vibration response and 7× ($N_{s} \omega$) and 14× ($2N_{s} \omega$) in the torsional vibration response. The mechanism will be deduced in detail in “Mechanism revealing and law explorating of vibration response”.

## Mechanism revealing and law explorating of vibration response

The above model based special case studies expounds the basic response characteristics of aerodynamic force and crack excitation. At the same time, it also shows that the structural form will significantly affect the response characteristics.

In a typical simplified system with five stationary blades (*N*_s_ = 5) and five moving blades (*N*_d_ = 5), the five moving blades are synchronously excited by the aerodynamic force (Synchronous excitation). In a typical simplified system with seven stationary blades (*N*_s_ = 7) and five moving blades (*N*_d_ = 5), the five moving blades are asynchronously excited by the aerodynamic force (asynchronous excitation), that is, each moving blade is excited by the aerodynamic force with a phase lag.

In this section, from the perspective of kinematics and dynamics, the internal response mechanism of synchronous excitation and asynchronous excitation are revealed first, and based on Number Theory, the general law of response characteristics of non-coprime forms (synchronous excitation) and coprime forms (asynchronous excitation) are obtained.

### Response mechanism under synchronous excitation

**Figure 10:**
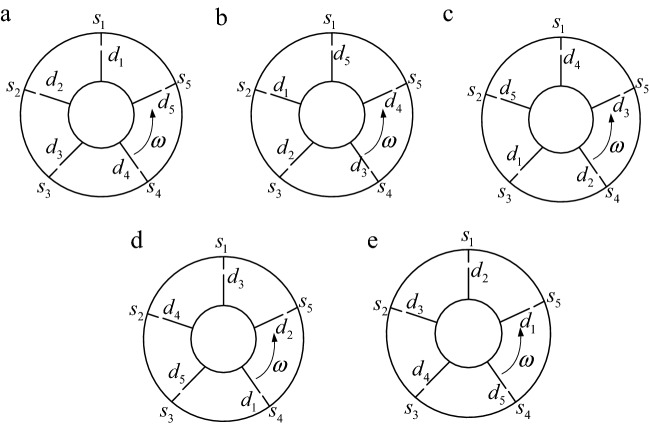
The position relationship of moving and stationary blades during one rotation cycle. (**a**) 0/5 cycle. (**b**) 1/5 cycle. (**c**) 2/5 cycle. (**d**) 3/5 cycle. (**e**) 4/5 cycle.

Figure 10 is the position relationship of moving and stationary blades during one rotation cycle (*N*_s_ = 5, *N*_d_ = 5). In the figure, *s*_*i*_ (*i* = 1, 2, 3, 4, 5) are the positions of stationary blades and *d*_*i*_ (*i* = 1, 2, 3, 4, 5) are the positions of moving blades.

**Figure 11:**
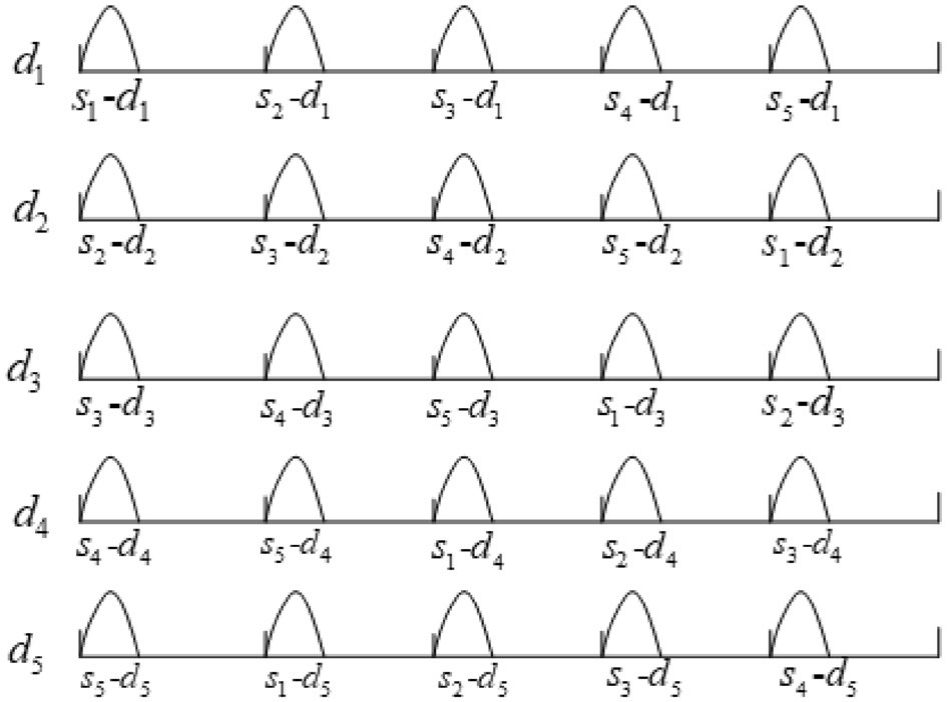
Excitation diagram of each moving blade during one rotation cycle.

Figure 11 is the excitation diagram of each moving blade during one rotation cycle, and *s*_i_–*d*_j_ represents the aerodynamic excitation of the *j*-th moving blade at the *i*-th stationary blade. When the numbers of moving and stationary blades are five, during one rotation cycle, due to the airflow at the upper stationary blades, each moving blade is excited by aerodynamic forces at five stationary blades successively, which means the fundamental frequency is $N_{s} \omega$, and the five moving blades are excited synchronously.

#### Torsional vibration

**Figure 12:**
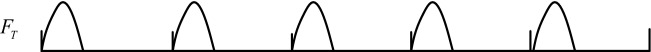
Torsional excitation diagram of shafting during one rotation cycle.

In an ideal and circularly symmetric blade disk rotor system, the excitation of the torsional vibration of the shaft is the superposition of the excitation torque of each moving blade with the same amplitude, frequency and phase. Therefore, the torsional vibration of the shaft is also equivalent to being excited by an excitation with the fundamental frequency $N_{s} \omega$, as shown in Fig. 12.

This is the mechanism why aerodynamic force causes $N_{s} \omega$, $2N_{s} \omega$ and $3N_{s} \omega$ frequency components in the torsional vibration response (shown in Fig. 7).

#### Transverse vibration

The excitation of transverse vibration is the superposition of the transverse components of the excitation of each moving blade. Because the moving blades are circularly symmetrical, the excitation amplitude A of each blade (i = 1, 2, …, *N*_*d*_) is equal for the ideal system. Therefore, the excitation of transverse vibration is the superposition of sinusoidal components with uniform phase lag $\left( {2\pi /N_{d} } \right)i$, which satisfies:

16 $$ A\sum\limits_{i = 1}^{{N_{d} }} {\sin \left( {\omega t + \frac{2\pi }{{N_{d} }}i} \right)} = 0 $$

This is the mechanism why the transverse vibration response amplitude is zero (shown in Fig. 6) under aerodynamic excitation but without crack and eccentric excitation.

When a blade has cracks, its coupling excitation effect on the shafting is different from that of other blades. Therefore, Eq. (16) is no longer equal to zeros, that is, the superposition of transverse excitation components of each moving blade is no longer zeros.

The mistuning excitation of a blade caused by a crack can be qualitatively expressed in the same form as the blade vibration $A\sin \left( {K_{j} N_{s} \omega t} \right) + C$. Moreover, since the cracked blade rotates with the rotating shaft, the transverse component of its mistuning excitation changes with the rotating frequency, which can be expressed as

17 $$ f_{c} = \sin \left( {\omega t} \right)\left( {A\sin \left( {K_{j} N_{s} \omega t} \right) + C} \right) $$

At this time, the transverse vibration has excitation with frequency components $\omega$ and $\left( {K_{j} N_{s} \pm 1} \right)\omega$, etc.

This explains why the amplitudes of the frequency component such as 1×, $\left( {N_{b} { - }1} \right)\omega$ = 4×, and $\left( {N_{b} { + }1} \right)\omega$ = 6× increase in transverse vibration under the coupling action of the crack and aerodynamic force, as shown in Fig. 6.

### Response mechanism under asynchronous excitation

This section will study another special case, the response mechanism with seven stationary blades and five moving blades. At this time, each moving blade is asynchronously excited by aerodynamic load (*N*_s_ = 7, *N*_d_ = 5).

**Figure 13:**
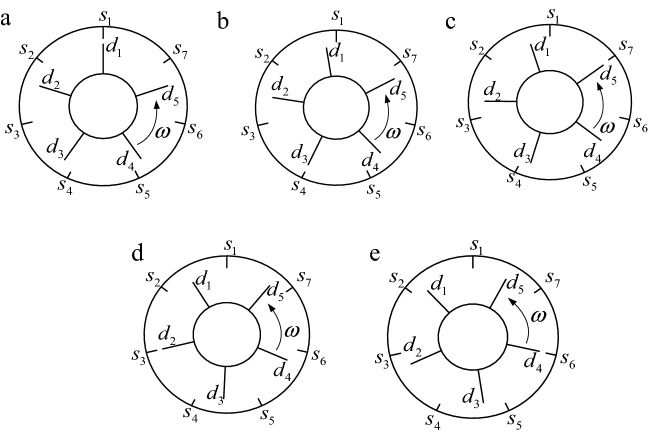
The position relationship of moving and stationary blades during the 1/*N*_s_ rotation cycle. (**a**) *d*_1_ forced:0/35 cycle. (**b**) *d*_3_ forced: 1/35 cycle. (**c**) *d*_5_ forced: 2/35 cycle. (**d**) *d*_2_ forced: 3/35 cycle. (**e**) *d*_4_ forced: 4/35 cycle.

Figure 13 shows the position relationship of moving and stationary blades during the 1/*N*_s_ rotation cycle. In the figure, *s*_*i*_ (*i* = 1, 2, 3, 4, 5, 6, 7) are the positions of stationary blades and *d*_*i*_ (*i* = 1, 2, 3, 4, 5) are the positions of moving blades. As can be seen in Fig. 13, during the 1/*N*_s_ rotation cycle, each moving blade is excited once in turn. Moreover, the excitation order of the moving blade is *d*_1_–> *d*_3_–> *d*_5_–> *d*_2_–> *d*_4_ (a)–> (b)–> (c)–> (d)–> (e)) and its phase lag is $\left( {1/N_{s} - 1/N_{d} } \right)2\pi$, that is 2*π*/35.

**Figure 14:**
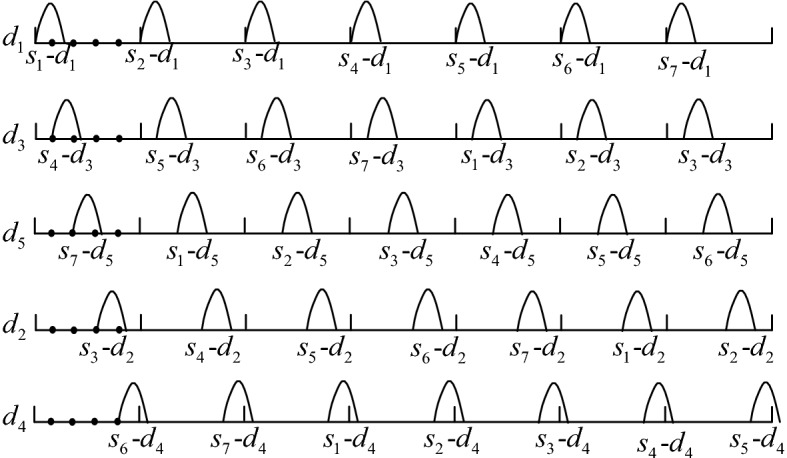
Excitation diagram of each moving blade during one rotation cycle.

Figure 14 shows a excitation diagram of each moving blade during one rotation cycle, and *s*_i_–*d*_j_ represents the aerodynamic excitation of the *j*-th moving blade at the *i*-th stationary blade. It can be seen in the figure that each moving blade is excited *N*_s_ times in one rotation cycle, and the phase lag of each excitation is 2*π*/*N*_s_.

#### Torsional vibration

**Figure 15:**
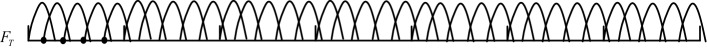
Torsional excitation diagram of shafting during one rotation cycle.

The excitation transmitted from the blade aerodynamic load to torsional vibration is the superposition of aerodynamic pulses with phase lag of each blade, and its schematic diagram is shown in Fig. 15. Therefore, the shaft torsional vibration is qualitatively excited by a load with a fundamental frequency $N_{s} \times N_{d}$.

Because the lag interval of each pulse cannot make each pulse completely independent, there is an overlapping area between pulses. Therefore, after the superposition of the pulses, the excitation pulsation amplitude decreases and the mean value increases,

Therefore, under the excitation of aerodynamic force, there are components with small amplitude and fundamental frequency of $N_{s} \times N_{d}$ in the torsional vibration response. That is the 35× frequency components are shown in Fig. 9.

When there is a crack on a blade, the torsional vibration excitation of the cracked blade to the shaft is not consistent with other normal blades, and there is an additional excitation component in torsional vibration caused by crack mistuning, with the blade vibration frequency $N_{s} \omega$ as the fundamental frequency. Therefore, under the coupling action of cracks and aerodynamic forces, 7× and 14× frequency components appear in the torsional vibration response, as shown in Fig. 9.

#### Transverse vibration

The aerodynamic force of each blade will cause excitation on the shaft. It can be seen in Fig. 14 that the shaft is excited $N_{s} \times N_{d}$ times in a rotation cycle, that is, the frequency of excitation is $N_{s} \times N_{d} \omega$. With the rotation of the shaft and the change in the excitation position, the transverse component of excitation changes periodically.

As can be seen in Fig. 13, the first excitation is that the moving blade *d*_1_ excited by aerodynamic force at *s*_1_ (Fig. 13a), and the second excitation is that the moving blade *d*_3_ excited by aerodynamic force at *s*_4_ (Fig. 13b). In this process, the shaft rotates by $\frac{2\pi }{{N_{s} N_{d} }}$ radians and the excitation position of the aerodynamic load rotates by $\frac{3}{{N_{s} }}2\pi$ radians (from *s*_1_ to *s*_4_). The position change laws of the subsequent excitation are the same.

Therefore, in this structural form, the change frequency of the excitation position has the following relationship with the shaft rotation frequency

18 $$ {{\left( {\frac{3}{{N_{s} }}2\pi } \right)} \mathord{\left/ {\vphantom {{\left( {\frac{3}{{N_{s} }}2\pi } \right)} {\frac{2\pi }{{N_{s} N_{d} }}}}} \right. \kern-\nulldelimiterspace} {\frac{2\pi }{{N_{s} N_{d} }}}}{ = 3}N_{d} $$

The transverse component of aerodynamic force changes with frequency $3N_{d} \omega$. Therefore, the transverse component of the aerodynamic force can be qualitatively expressed as

19 $$ f_{a} = A\sin (3N_{d} \omega t)\left( {\sin \;(N_{s} \times N_{d} \omega t) + C} \right) $$

By expanding the above formula, it can be seen that the frequency components of transverse excitation are $3N_{d} \omega$, $\left( {N_{s} \pm 3} \right) \times N_{d} \omega$, etc.

This explains why the amplitudes of the frequency component such as $3N_{d} \omega$ = 15× and $\left( {N_{s} { - }3} \right) \times N_{d} \omega$ = 20×, increase in transverse vibration under aerodynamic force, as shown in Fig. 8.

When there is a crack in a blade, the frequency component of the additional excitation caused by crack mistuning is the blade vibration frequency $KN_{s} \omega$, and the of transverse component on the shaft changes with the frequency $\omega$. Therefore, the transverse component caused by crack mistuning can be expressed as $A\sin \left( {\omega t} \right)\left( {\sin \left( {KN_{s} \omega t} \right) + C} \right)$.

This explains why the amplitudes of the frequency component such as $\left( {N_{s} - 1} \right)\omega$ = 6×, and $\left( {N_{s} + 1} \right)\omega$ = 8×, increase in transverse vibration under the coupling action of the crack and aerodynamic force, as shown in Fig. 8.

### General law of different structural forms

Special case studies of two typical structural forms show that the response characteristics of aerodynamic force and crack misuning and the mechanism of excitation are significantly different under different structural forms. Going a step further, this section will establish the response law of general structural form based on number theory.

The structural forms can be divided into two categories: in the first category, the numbers of moving blades and stationary blades are **coprime**, that is, there is no common divisor other than 1; in the second category, the numbers of moving blades and stationary blades are **non-coprime**, that is, there is a common divisor other than 1.

#### Coprime structural form

For a system where the number of moving and stationary blades are coprime, no more than one moving blade is excited by aerodynamic force at any time, and the excitation of each moving blade has phase lag, i.e., asynchronous excitation, such as the typical special case in “Response characteristics of asynchronous excitation” and “Response mechanism under asynchronous excitation”, where *N*_*s*_ = 7 and *N*_*d*_ = 5.

When *N*_*s*_ and *N*_*d*_ are coprime, the general response law can be revealed by Bézout’s identity

If the integers a and b have the greatest common factor d, Then, there must be integers i and j to make ai + bj = d.

According to the above theorem, when *N*_*s*_ and *N*_*d*_ are coprimes, their greatest common factor *d* = 1. Therefore, there must be integers *i* and *j* to make $N_{d} j + N_{s} i = 1$. Considering that *i* is an arbitrary integer, there must also be an integer *i* to make $N_{d} j - N_{s} i = 1$.

Divide $N_{d} N_{s}$ on both sides of the above formula to obtain

20 $$ \frac{j}{{N_{s} }} - \frac{i}{{N_{d} }} = \frac{1}{{N_{d} N_{s} }} $$

and

21 $$ \frac{j}{{N_{s} }} = \frac{i}{{N_{d} }} + \frac{1}{{N_{d} N_{s} }} $$

Multiply $2\pi$ on both sides to obtain

22 $$ \frac{j}{{N_{s} }}2\pi = \frac{i}{{N_{d} }}2\pi + \frac{1}{{N_{d} N_{s} }}2\pi $$

The above formula shows that when *N*_*s*_ and *N*_*d*_ are coprime and the serial number of the coincident moving and stationary blades is set to 0, the angle difference between the *i*-th moving blade and the *j*-th stationary blade is $\frac{1}{{N_{d} N_{s} }}2\pi$.

Therefore, during the rotation of the shaft, the next *i*-th moving blade can coincide with the next *j*-th stationary blade passing through the $\frac{1}{{N_{d} N_{s} }}2\pi$ radian and be excited by aerodynamic force.

In this process, the shaft rotates by $\frac{2\pi }{{N_{s} N_{d} }}$ radians and the excitation position of the aerodynamic load rotates by $\frac{j}{{N_{s} }}2\pi$ radians. Therefore, the change frequency of the excitation position has the following relationship with the shaft rotation frequency:

23 $$ {{\left( {\frac{j}{{N_{s} }}2\pi } \right)} \mathord{\left/ {\vphantom {{\left( {\frac{j}{{N_{s} }}2\pi } \right)} {\left( {\frac{1}{{N_{d} N_{s} }}2\pi } \right)}}} \right. \kern-\nulldelimiterspace} {\left( {\frac{1}{{N_{d} N_{s} }}2\pi } \right)}} = N_{d} j $$

The transverse component of aerodynamic force changes with frequency $N_{d} j\omega$. Therefore, the transverse component of the aerodynamic force can be qualitatively expressed as

24 $$ f_{a} = A\sin \left( {N_{d} j\omega t} \right)\left( {\sin \left( {N_{s} N_{d} \omega t} \right) + C} \right) $$

The excitation transmitted from the blade aerodynamic force to torsional vibration is the superposition of aerodynamic pulses with phase the lag of each blade. Therefore, the shaft torsional vibration is qualitatively excited by a load with a fundamental frequency $N_{s} \times N_{d}$. Therefore, the excitation of torsional vibration can be qualitatively expressed as $A\sin \left( {N_{s} N_{d} \omega t} \right) + C$.

Specifically, in “Response mechanism under asynchronous excitation”, when *N*_s_ = 7 and *N*_d_ = 5, *j* = 3 and *i* = 2,

25 $$ \frac{3}{{N_{s} }}2\pi = \frac{2}{{N_{d} }}2\pi { + }\frac{1}{{N_{d} N_{s} }}2\pi $$

Therefore, in Fig. 13, the next excitation positions are the next 2-th moving blades (*d*_1_–> *d*_3_–> *d*_5_–> *d*_2_–> *d*_4_) passing through the next 3-th stationary blades (*s*_1_–> *s*_4_–> *s*_7_–> *s*_3_–> *s*_6_).

#### Non-coprime structural form

For a system where the number of moving and stationary blades are coprimes, that is, there is a maximum common divisor *m* (m ≠ 1) between *N*_*s*_ and *N*_*d*_. The results show that at any excitation position, there is a group of circularly symmetrical moving blades with a number of *m*, which are synchronously excited by aerodynamic force.

According to Eq. (16), the resultant force of the transverse components of a group of cyclic symmetry blades under synchronous excitation is zero. Therefore, under the excitation of aerodynamic force, the response amplitude of the non-coprime ideal system is zero.

Since the *m* moving blades are excited at the same time, the torsional excitation of the *m* blades is superimposed to form a whole excitation. Therefore, in one rotation cycle, the torsional excitation frequency is $N_{s} N_{d} \omega /m$. Therefore, torsional excitation can be qualitatively expressed as:

26 $$ F_{T} = A\sin \left( {\left( {N_{s} N_{d} \omega /m} \right)t} \right) + C $$

#### Crack characteristics

When there is a crack in a blade, the frequency component of the additional excitation caused by crack mistuning is the blade vibration frequency $KN_{s} \omega$. Therefore, the additional excitation component in torsional vibration caused by crack mistuning can be expressed as $\sin \left( {KN_{s} \omega t} \right) + C$. Considering that the transverse component of the excitation of the cracked moving blade changes with the frequency $\omega$, the transverse component caused by crack mistuning can be expressed as $A\sin \left( {\omega t} \right)\left( {\sin \left( {KN_{s} \omega t} \right) + C} \right)$.

## Conclusions

In this paper, the typical frequency characteristics of the vibration response for two structural forms under the actions of aerodynamic force and blade cracks are obtained by numerical solutions. Then, in view of why such frequency characteristics occur, we adopt the principles of kinematics and dynamics to reveal the internal mechanisms between the vibration responses and the excitations. Furthermore, generally, we adopt number theory to establish the general laws of responses with general structural forms. The conclusions are as follows.

1. For a system in which the numbers of moving blades and stationary blades are coprime, the moving blades are asynchronously excited by the aerodynamic force at each stationary blade, successively. The general form of excitation for lateral vibration by aerodynamic loading is $A\sin \left( {N_{d} j\omega t} \right)\left( {\sin \left( {N_{s} N_{d} \omega t} \right) + C} \right)$ and the general form of excitation for torsional vibration by aerodynamic loading is $A\sin \left( {N_{s} N_{d} \omega t} \right) + C$.
2. For a system in which the numbers of moving blades and stationary blades are non-coprime (the common divisor is *m*), there are *m* moving blades with cyclic symmetrical distribution excited by aerodynamic force. The excitation superposition result of transverse components of these *m* blades is zero. Therefore, there is no excitation in lateral vibration. The general form of excitation for torsional vibration by aerodynamic loading is $A\sin \left( {\left( {N_{s} N_{d} \omega /m} \right)t} \right) + C$.
3. When there is a crack in a moving blade, the vibration response of the cracked blade under aerodynamic loading is different from that of other blades, and the stiffness parameter excitation of the crack appears. Therefore, the cracked blade will causes new excitation into the torsional vibration and transverse vibration of the system. In torsional vibraton, the amplitudes corresponding to frequency components $K_{j} N_{s} \omega$ are increased. In the lateral vibration, new modulation components are produced with the blade vibration frequency as the carrier and the rotation frequency as the modulation source $A\sin \left( {\omega t} \right)\left( {\sin \left( {K_{j} N_{s} \omega t} \right) + C} \right).$
4. At present, this study only focuses on the ideal and circularly symmetric blade disk rotor system, and inevitable random mistuning is not considered. The complex coupling characteristics and mechanism between mistuned moving blades, mistuned static blades and inherent eccentricity will be discussed in a future paper.

## Supplementary Information

Supplementary Information.

## Data Availability

All data generated or analyzed during this study are included in this published article.
