# Supplementary material for: The vibration response mechanism of a blade disk rotor system under the coupling effects of cracks and aerodynamic forces
Source: Sci Rep. 2022 Jan 27;12:1520. doi: 10.1038/s41598-022-05543-x (PMC8795153; doi:10.1038/s41598-022-05543-x)
Supplement: Supplementary file 1 — Supplementary Information. [file 41598_2022_5543_MOESM1_ESM.doc]

**Appendix A :** The detail expressions of absolute coordinates:

$z_{Q}=-r\cos(\Omega t+\theta)\frac{\partial u_{x}(z_{d},t)}{\partial z}-r\sin(\Omega t+\theta)\frac{\partial u_{y}(z_{d},t)}{\partial z}$ (A1)

(A2)

(A3)

$\begin{matrix} z_{D}=-(l+r_{2})\sin(\Omega t+\theta_{bi})\frac{\partial u_{y}(z_{d},t)}{\partial z}-(l+r_{2})\cos(\Omega t+\theta_{bi})\frac{\partial u_{x}(z_{d},t)}{\partial z} \\ +v(l,t)\sin\left(\beta\right) \end{matrix}$ (A4)

(A5)

(A6)

**Appendix B:** The governing equations of the cracked blade-disk-shaft system.

(1) Equation expressions associated with the generalized coordinates $\mathbf{q}_{x}$:

(B1)

where：

$$\mathbf{M}_{x}=\rho_{s}A_{s}\int_{0}^{s} \mathbf{UU}^{T}\mathrm{d}z$$

$$\mathbf{K}_{x}=E_{s}I_{sx}\int_{0}^{s} \frac{\partial^{2}\mathbf{U}}{\partial z^{2}}\frac{\partial^{2}\mathbf{U}^{T}}{\partial z^{2}}\mathrm{d}z$$

$$\mathbf{M}_{dxzd}=\rho_{d}h_{d}\pi\left(r_{2}^{2}-r_{1}^{2}\right)\left.\mathbf{UU}^{T}\right|_{z_{d}}$$

$$\mathbf{M}_{dxdzd}=\frac{1}{4}\rho_{d}h_{d}\pi\left(r_{2}^{4}-r_{1}^{4}\right)\left.\frac{\partial\mathbf{U}}{\partial z}\frac{\partial\mathbf{U}^{T}}{\partial z}\right|_{z_{d}}$$

$$\mathbf{M}_{vxdzd}=\rho_{b}A_{b}\left(\frac{1}{3}L^{3}+r_{2}L^{2}+r_{2}^{2}L\right)N\left.\frac{\partial\mathbf{U}}{\partial z}\frac{\partial\mathbf{U}^{T}}{\partial z}\right|_{z_{d}}$$

$$\mathbf{M}_{vxdzdv}=\rho_{b}A_{b}\sin\left(\beta_{b}\right)\left.\frac{\partial\mathbf{U}}{\partial z}\right|_{z_{d}}\int_{0}^{L} (l+r_{2})\mathbf{V}^{T}\mathrm{d}l$$

$$\mathbf{M}_{vxzd}\text{=}\rho_{b}A_{b}LN\left.\mathbf{UU}^{T}\right|_{z_{d}}$$

$$\mathbf{M}_{vxzdv}\text{=}\rho_{b}A_{b}\cos\left(\beta_{b}\right)\left.\mathbf{U}\right|_{z_{d}}\int_{0}^{L} \mathbf{V}^{T}\mathrm{d}l$$

$$\mathbf{C}_{x}=\alpha\mathbf{M}_{x}+\beta\mathbf{K}_{x}$$

(2) Equation expressions associated with the generalized coordinates $\mathbf{q}_{y}$:

( B2)

where:

$$\mathbf{M}_{y}=\rho_{s}A_{s}\int_{0}^{s} \mathbf{UU}^{T}\mathrm{d}z$$

$$\mathbf{K}_{y}=E_{s}I_{sx}\int_{0}^{s} \frac{\partial^{2}\mathbf{U}}{\partial z^{2}}\frac{\partial^{2}\mathbf{U}^{T}}{\partial z^{2}}\mathrm{d}z$$

$$\mathbf{M}_{dyzd}=\rho_{d}h_{d}\pi\left(r_{2}^{2}-r_{1}^{2}\right)\left.\mathbf{UU}^{T}\right|_{z_{d}}$$

$$\mathbf{M}_{dydzd}=\frac{1}{4}\rho_{d}h_{d}\pi\left(r_{2}^{4}-r_{1}^{4}\right)\left.\frac{\partial\mathbf{U}}{\partial z}\frac{\partial\mathbf{U}^{T}}{\partial z}\right|_{z_{d}}$$

$$\mathbf{M}_{vydzd}=\rho_{b}A_{b}\left(\frac{1}{3}L^{3}+r_{2}L^{2}+r_{2}^{2}L\right)N\left.\frac{\partial\mathbf{U}}{\partial z}\frac{\partial\mathbf{U}^{T}}{\partial z}\right|_{z_{d}}$$

$$\mathbf{M}_{vydzdv}=\rho_{b}A_{b}\sin\left(\beta_{b}\right)\left.\frac{\partial\mathbf{U}}{\partial z}\right|_{z_{d}}\int_{0}^{L} (l+r_{2})\mathbf{V}^{T}\mathrm{d}l$$

$$\mathbf{M}_{vyzd}\text{=}\rho_{b}A_{b}LN\left.\mathbf{UU}^{T}\right|_{z_{d}}$$

$$\mathbf{M}_{vyzdv}\text{=}\rho_{b}A_{b}\cos\left(\beta_{b}\right)\left.\mathbf{U}\right|_{z_{d}}\int_{0}^{L} \mathbf{V}^{T}\mathrm{d}l$$

$$\mathbf{C}_{y}=\alpha\mathbf{M}_{y}+\beta\mathbf{K}_{y}$$

(3) Equation expressions associated with the generalized coordinates :

(B3)

where:

(4) Equation expressions associated with the generalized coordinates $\mathbf{q}_{v}$:

(B4)

where:

$$\mathbf{M}_{v}=\rho_{b}A_{b}\int_{0}^{L} \mathbf{VV}^{T}\mathrm{d}l$$

$$\mathbf{M}_{svdzds}=\rho_{b}A_{b}\sin\left(\beta_{b}\right)\left.\frac{\partial\mathbf{U}}{\partial z}\right|_{z_{d}}\int_{0}^{L} (l+r_{2})\mathbf{V}^{T}\mathrm{d}l$$

$$\mathbf{M}_{sdvzds}=\rho_{b}A_{b}\cos\left(\beta_{b}\right)\left.\mathbf{U}\right|_{z_{d}}\int_{0}^{L} \mathbf{V}^{T}\mathrm{d}l$$

$$\mathbf{K}_{v}=EI\int_{0}^{L} \frac{\partial^{2}\mathbf{V}}{\partial l^{2}}\frac{\partial^{2}\mathbf{V}^{T}}{\partial l^{2}}\mathrm{d}l$$

$$\mathbf{K}_{\Omega}\text{=}\frac{1}{2}\rho A_{b}\int_{0}^{L} [(r_{2}+L)^{2}-\left(r_{2}+l\right)^{2}]\frac{\partial\mathbf{V}}{\partial l}\frac{\partial\mathbf{V}^{T}}{\partial l}\mathrm{d}l$$

$$\mathbf{K}_{\text{cp}}\text{=}\int_{0}^{L} k_{\text{cp}}(l)\left(\frac{\partial\mathbf{V}}{\partial l}\frac{\partial\mathbf{V}^{T}}{\partial l}\right)\mathrm{d}l$$

$$\mathbf{C}_{v}=\alpha\mathbf{M}_{v}+\beta\mathbf{K}_{v}$$

(5) Equation expressions associated with the cracked blades:

(B5)

where:

$K_{c}=\frac{(EI)^{2}}{K_{crack}}V(l_{c})^{n}V(l_{c})^{nT}$.

(6) Generalized force matrix

$Q_{i}\text{=}\int_{\xi_{0}}^{\xi_{1}} \left[f\left(\xi,t\right)\psi_{i}(\xi)+M\left(\xi,t\right)\psi'_{i}(\xi)\right]d\xi$ (B6)

$f\left(\xi,t\right)$—Force in physical space

$M\left(\xi,t\right)$—Moment in physical space

$\psi_{i}(\xi)$—Assumed mode

$\xi_{0}$, $\xi_{1}$—The geometric boundary of the corresponding component.

**Appendix C : Model parameters**.

Table C1. Model parameters

| Symbols | Physical meaning | Value |
| --- | --- | --- |
| $L$ | Length of blade | 0.06 m |
| $b$ | Width of blade | 0.125 *L* |
| $h_{b}$ | Thickness of blade | 0.2 *b* |
| $\rho_{b}$ | Density of blade | 7900 kg/m^3^ |
| $\beta$ | Setting angle of blade | 0° |
| $\theta_{bi}$ | Reference angle of blade | $i\frac{2\pi}{N}, i=0,1,\cdots N_{j}-1$ |
| $\rho_{d}$ | Density of disk | 7900 kg/m^3^ |
| $h_{d}$ | Thickness of disk | $b$ |
| $r_{2}$ | External diameter of disk | *L/2* |
| $r_{1}$ | Internal diameter of disk | *L/12* |
| $r_{s}$ | Radius of shaft | $r_{1}$ |
| $S$ | Length of shaft | 10 *L* |
| $z_{d}$ | Axial position of disk | 0.4*S* |
| $E_{s}$ | Young's modulus | 200Gpa |
| $\mu$ | Poisson ratio | 0.3 |
| $\rho_{s}$ | Density of shaft | 7900 kg/m^3^ |
